# Population Genomics of Pooled Samples: Unveiling Symbiont Infrapopulation Diversity and Host–Symbiont Coevolution

**DOI:** 10.3390/life13102054

**Published:** 2023-10-14

**Authors:** Alix E. Matthews, Than J. Boves, Katie L. Percy, Wendy M. Schelsky, Asela J. Wijeratne

**Affiliations:** 1 College of Sciences and Mathematics and Molecular Biosciences Program, Arkansas State University, Jonesboro, AR 72401, USA; 2 Department of Biological Sciences, Arkansas State University, Jonesboro, AR 72401, USA; tboves@astate.edu (T.J.B.); awijeratne@astate.edu (A.J.W.); 3 Audubon Delta, National Audubon Society, Baton Rouge, LA 70808, USA; katie.percy@usda.gov; 4 United States Department of Agriculture, Natural Resources Conservation Service, Addis, LA 70710, USA; 5 Department of Evolution, Ecology, and Behavior, School of Integrative Biology, University of Illinois, Urbana-Champaign, Champaign, IL 61801, USA; schelsky@illinois.edu; 6 Prairie Research Institute, Illinois Natural History Survey, University of Illinois, Urbana-Champaign, Champaign, IL 61820, USA

**Keywords:** Astigmata, diversity, ectosymbionts, feather mites, high-throughput sequencing, low-input DNA

## Abstract

Microscopic symbionts represent crucial links in biological communities. However, they present technical challenges in high-throughput sequencing (HTS) studies due to their small size and minimal high-quality DNA yields, hindering our understanding of host–symbiont coevolution at microevolutionary and macroevolutionary scales. One approach to overcome those barriers is to pool multiple individuals from the same infrapopulation (i.e., individual host) and sequence them together (Pool-Seq), but individual-level information is then compromised. To simultaneously address both issues (i.e., minimal DNA yields and loss of individual-level information), we implemented a strategic Pool-Seq approach to assess variation in sequencing performance and categorize genetic diversity (single nucleotide polymorphisms (SNPs)) at both the individual-level and infrapopulation-level for microscopic feather mites. To do so, we collected feathers harboring mites (Proctophyllodidae: *Amerodectes protonotaria*) from four individual Prothonotary Warblers (Parulidae: *Protonotaria citrea*). From each of the four hosts (i.e., four mite infrapopulations), we conducted whole-genome sequencing on three extraction pools consisting of different numbers of mites (1 mite, 5 mites, and 20 mites). We found that samples containing pools of multiple mites had more sequencing reads map to the feather mite reference genome than did the samples containing only a single mite. Mite infrapopulations were primarily genetically structured by their associated individual hosts (not pool size) and the majority of SNPs were shared by all pools within an infrapopulation. Together, these results suggest that the patterns observed are driven by evolutionary processes occurring at the infrapopulation level and are not technical signals due to pool size. In total, despite the challenges presented by microscopic symbionts in HTS studies, this work highlights the value of both individual-level and infrapopulation-level sequencing toward our understanding of host–symbiont coevolution at multiple evolutionary scales.

## 1. Introduction

Symbiotic relationships—broadly defined as two different species living in close physical or physiological association with one another—are among the most common interactions in the natural world [1]. These intimate interactions play a fundamental role in an individual’s health [2], in biological community structure [3], and in the entire functioning of ecosystems [4]. Consequently, symbionts (i.e., organisms that form parasitic, mutualistic, or commensal interactions with hosts [5]) have strong effects on biodiversity, and symbionts themselves comprise the majority of species on Earth [6,7]. Despite their ubiquity and importance in the natural world, basic biological knowledge about many symbionts is still lacking, including their ecology and coevolutionary history with hosts.

Technical challenges associated with studying symbionts add to these knowledge gaps and can consequently hinder our understanding of symbionts and their relationships with hosts at multiple evolutionary scales. One major limitation is that there are few taxonomic and morphological experts for most groups of symbionts, so basic research on species identification, description, and biodiversity is subjected to a severe bottleneck [8,9]. Fortunately, the field of molecular genetics has helped overcome some of the taxonomic expertise limitations and has aided in rapid species discovery and augmented species identification [10,11]. In particular, the application of whole-genome high-throughput sequencing (HTS) has transformed our understanding of host–symbiont macroevolutionary processes and patterns. For example, coevolutionary studies applying whole-genome HTS have unveiled major historical host-switching events between distantly related taxa, which has revolutionized our understanding of symbiont diversification and geographic distribution [12,13]. The application of HTS has also allowed us to explore genomic underpinnings of the variation (or lack thereof) in symbiont host specificity [14,15], providing us fine-scale insight into symbiont–host ecology and coevolution. However, genome-wide HTS studies for microscopic symbionts are often limited by the minimal amount of high-quality DNA that can be extracted for downstream use [16]. The lack of high-quality reference genomes for non-model organisms (as is the case with many undescribed symbionts [17]) further complicates genome-wide investigations. These challenges have not only limited our understanding of macroevolutionary patterns, but also the microevolutionary processes that drive those patterns, such as dispersal and gene flow.

One approach to surpass the barrier of low DNA yield/quality for microscopic symbionts in a HTS context is to pool multiple individuals into a single DNA extraction prior to sequencing (i.e., pooled sequencing or Pool-Seq). This approach can be strategically implemented based on budget, goals of the study, and biology of the system [18]. In the case of symbionts, individuals from the same population on an individual host (i.e., infrapopulation or deme) can be pooled and sequenced together. Genomic information gleaned from HTS at the infrapopulation level can provide insight not only on processes occurring on a much finer scale, such as natural selection and genetic drift, but also about the role of life history and ecology in those processes from both the symbiont and host perspectives [19]. However, valuable information about an individual’s genetic contribution to the infrapopulation is lost with Pool-Seq [20]. To address this issue, a strategic pooled-sequencing approach can be applied to explore both individual-level and infrapopulation-level diversity simultaneously, such that individuals from the same infrapopulation can be sequenced in different sized pools (e.g., 1–50 individuals from a single infrapopulation). Variations of this approach have been applied to assess the accuracy and cost-effectiveness of individual sequencing versus pooled sequencing in both model (e.g., *Drosophila melanogaster* [21]; *Apis mellifera* [22]) and non-model (e.g., pine processionary moth, *Thaumetopoea pityocampa* [23]) species. These studies have established that pooled sequencing is a comparable approach to individual sequencing and can accurately (and cost-effectively) capture the genetic diversity of a population. However, this type of pooled HTS approach has not been applied in a host–symbiont context, where it may provide valuable insight regarding the coevolution of symbioses at both the individual and infrapopulation levels.

Vane-dwelling feather mites (Acari: Acariformes: Astigmata: Analgoidea, Pterolichoidea) represent an ideal system with which to implement both individual-level and infrapopulation-level sequencing. Feather mites are ubiquitous, obligate symbionts of birds [24,25] with variable infrapopulation sizes [26,27,28]. Although they are dispersal-limited and typically vertically transmitted [29,30], macroevolutionary (i.e., species level) studies have demonstrated that they often do not perfectly coevolve with their hosts. Instead, there is evidence for clade-limited host switching and a wide range of host specificity, which could be associated with the differential life histories of mites [31] or hosts [32,33,34]. Microevolutionary studies (i.e., infrapopulation level) are rare and have been restricted to barcoding markers [35,36], limiting our understanding of their infrapopulation genetic diversity to a small number of conserved loci instead of genome-wide. This is at least partly due to their minute size (0.3–0.7 mm in length [37]), which makes obtaining sufficient amounts of high-quality DNA for whole-genome HTS endeavors challenging, particularly for individual mites. As a result, individual-level and infrapopulation-level feather mite diversity and the importance of that diversity with respect to coevolution with their hosts remain largely underexplored.

In this study, we investigated the individual and infrapopulation genetic diversity of a symbiotic feather mite species, *Amerodectes protonotaria* Hernandes 2018 (Astigmata: Proctophyllodidae), through a pooled HTS approach. *Amerodectes protonotaria* is a host-specialist mite found thus far only on *Protonotaria citrea* Boddaert 1783 (Prothonotary Warbler; Parulidae) [28,33,38,39] and is the only feather mite species with a fully sequenced and assembled genome [40]. We sequenced DNA from individual mites as well as various sized pools of mites from the same infrapopulation and then compared the (1) sequencing and read-mapping performance on low-input samples obtained from the different amounts of tissue (i.e., different numbers of individual mites) and (2) genetic diversity across and within datasets. We then discussed the insights gleaned from these different datasets with respect to basic biology, ecology, and (co)evolution of small symbiotic organisms such as feather mites, as well as the advantages and disadvantages of applying these techniques in (co)evolutionary studies.

## 2. Materials and Methods

### 2.1. Field Collections

We collected *Amerodectes protonotaria* from four adult male Prothonotary Warblers in Illinois (Big Cypress Access Nature Preserve; *n* = 2) and Louisiana (BREC’s Bluebonnet Swamp Nature Center; *n* = 2), USA. All birds were captured during the breeding season with mist nets using audio playback lures. We collected one to two tail feathers harboring mites and placed them in individually labeled containers. Mites were later isolated from feathers under a stereomicroscope and preserved in 95% ethyl alcohol at −20 °C until DNA extraction. Birds were captured and ethically handled under the necessary and appropriate federal and local permits (see Acknowledgements). Field collection data are listed in Table S1.

### 2.2. DNA Extraction, Library Preparation, and Sequencing

We conducted three genomic DNA (gDNA) isolations of mites per individual host (i.e., infrapopulation of mites) for a total of 12 isolations. From each infrapopulation, we randomly selected mites to be added to different sized gDNA isolation pools. Specifically, from each of the four infrapopulations, we isolated gDNA from: (1) a single mite (one female); (2) five mites pooled together (three females and two males); and (3) 20 mites pooled together (15 females and five males). Although lower than the recommended pool size of 40 individuals [18], we wanted to determine the lowest ranges of input tissue needed for whole-genome sequencing experiments (e.g., microscopic symbionts). Generally, around 0.5 ng of gDNA can be obtained from a single (female) *A. protonotaria* extraction (A.E.M., personal observations). We used the QIAamp DNA Micro Kit (QIAGEN, Germantown, MD, USA) tissue sample protocol, with slight modifications that have been previously described [33,38]. Briefly, our modifications included the following: using a sterile mounting needle to pierce a minute hole in the mite exoskeletons (for the samples containing one and five mites) or using a small sterile pestle to crush mites (for the samples containing 20 mites) while in lysis buffer; instead of vortexing samples, we gently pipetted and/or inverted them; for elution, we preheated the elution buffer and extended both the incubation and centrifugation steps.

Library construction and sequencing were carried out at the Roy J. Carver Biotechnology Center, University of Illinois at Urbana-Champaign. The shotgun libraries were constructed from up to 5 ng of DNA after sonication with a Covaris ME220 sonicator (Covaris Inc., Woburn, MA, USA) to an average fragment size of 400 bp using the UtraLow-Input DNA Library Construction kit (Tecan, Morrisville, NC, USA). The dual-indexed libraries were amplified with 8 cycles of PCR and run on a Fragment Analyzer (Agilent, Santa Clara, CA, USA). Libraries were pooled in equimolar concentration and further quantitated via qPCR on a BioRad CFX Connect Real-Time System (Bio-Rad Laboratories, Hercules, CA, USA). The libraries were loaded on an Illumina NovaSeq 6000 SP lane and sequenced for 151 cycles from both ends of the fragments (i.e., paired-end sequencing). The fastq read files were generated and demultiplexed with the bcl2fastq v2.20 Conversion Software (Illumina, San Diego, CA, USA). Raw reads were deposited to the NCBI SRA database (Table S1).

### 2.3. Sequence Quality Control and Species Validation

Demultiplexed reads were trimmed of Illumina sequencing adapters first using cutadapt version 3.0 [41] with a Phred score quality threshold of 30. Using BBDuk (https://sourceforge.net/projects/bbmap/, accessed on 6 September 2021), we further trimmed the 3′ end of resulting reads to 140 bases and removed duplicate reads. The resulting reads were then analyzed using FastQC version 0.11.5 (http://www.bioinformatics.babraham.ac.uk/projects/fastqc/, accessed on 6 September 2021).

To confirm that the mites isolated from feathers were indeed *Amerodectes protonotaria*, we used the automated Target Restricted Assembly Method (aTRAM) version 2.3.4 [42] to target and assemble the cytochrome c oxidase subunit 1 (*cox1*) gene from our quality-controlled reads. We first conducted a BLASTn on the resulting *cox1* sequences to confirm that samples had high sequence similarity to *A. protonotaria*. All but one sample (PROW953_R1; 1 mite sample from PROW953) had high sequence similarity with *A. protonotaria* (>99% identity over 1190 bp); PROW953_R1 had high sequence similarity to multiple bacteria of the family Burkholderiaceae. For this sample, we mapped reads to the top three bacterial matches using BWA MEM [43]. We extracted the unmapped (i.e., non-bacterial) reads for use in aTRAM; after removing these bacterial reads, the top *cox1* BLASTn result was *A. protonotaria* for this sample (>99% identity over 1190 bp). We then aligned the *cox1* sequences from all 12 samples sequenced herein along with previously curated *cox1* sequences of *A. protonotaria* and other closely related (other *Amerodectes* spp. and a *Tyrannidectes* sp., from the same subfamily as *Amerodectes* (Pterodectinae)) and distantly related (*Proctophyllodes* spp., from a different subfamily in Proctophyllodidae (Proctophyllodinae) and a *Trouessartia* sp., from a different family of feather mites (Trouessartiidae)) feather mite lineages collected from parulid warblers [33,38]. We aligned sequences in MAFFT version 7.503 [44] and estimated a maximum likelihood (ML) phylogeny in IQ-Tree version 1.6.12 [45] and ModelFinder [46] with 1000 ultrafast bootstrap replicates as measures of branch support. We confirmed species identity if samples fell clearly within the *A. protonotaria* clade.

### 2.4. Read Mapping and Variant Detection

High-quality reads were mapped to the *A. protonotaria* draft reference genome (PROW981_R1; the single-mite sample from PROW981 in this study [40]) using BWA MEM and sorted using SAMtools version 1.13 [47]. As a read-mapping comparison, we also mapped high-quality reads for all samples to three Burkholderiaceae genomes, which were highly abundant in one sample (see previous section). We marked PCR duplicates using picard tools version 2.17.10 within the Genome Analysis Toolkit (GATK) version 4.2.6.1 [48]. We applied several hard filters to the resulting reads. We first removed low-quality mapped reads by skipping alignments with a MAPQ score < 20 in SAMtools version 1.15.1. We then calculated the average read depth for each species (~15X) using SAMtools to determine an appropriate maximum read depth (50) for generating mpileup files using BCFtools version 1.15.1 [47].

We conducted joint genotyping to call SNPs on the resulting mpileup files using BCFtools. We excluded low-genotyping-quality sites (<30) and sites that were different from the reference but equal to each other. We included a minor allele frequency threshold of 0.05 and excluded sites that had depth <10 using BCFTools. We excluded sites that were not present in 100% of individuals (i.e., we allowed 0% missing data) using vcftools version 0.1.15 [49]. Although linkage is difficult to estimate for Pool-Seq data given the loss of haplotype information [18], we removed potentially linked SNPs above two contrasting r^2^ thresholds (0.01 and 0.99) with sliding windows of 100 SNPs using a step size of 50 in PLINK version 1.90b5.2 [50] as a comparative exercise to explore the effect on our results. Many other analytical frameworks exist for inferring different population genetic statistics from Pool-Seq data [51,52,53] with variable benchmarking results [54,55], which alludes to the difficulty of untangling Pool-Seq data [18,23,56]. Our strategy described above is highly conservative, utilizes well-maintained public programs, and allows us to more directly compare population structure [57]. We visually assessed the impact of the different linkage thresholds through principal components analyses (PCA) using PLINK. PCAs were visualized in R version 3.6.3 [58] using tidyverse version 3.1 [59], ggplot2 version 3.3.5 [60], and ggpubr version 0.4.0 [61]. We used sambamba version 0.8.2 [62] to calculate the read depth at each SNP site for both linkage thresholds. We also explored how the total number of SNPs varied within and between samples under the different linkage filtering parameters. Lastly, UpSet plots [63] were created in ggupsetr version 0.3.0 [64] to visualize how many SNPs were shared within and between samples and to identify which SNPs were shared.

As a preliminary analysis to explore the potential function of the genes in which SNPs are located, we conducted a BLASTx on FlyBase [65] against the well-annotated genome database of *Drosophila melanogaster* (Insecta: Drosophilidae). We used the UpSet plot matrix interactions to identify SNP sites that were common among all samples (*n* = 6) under both linkage thresholds. Our BLASTx queries were regions of our reference genome that consisted of 100 bp upstream and 100 bp downstream of each SNP site (201 bp total). We investigated the top hit polypeptides based on the lowest bit scores and E-values. From these BLAST hits and associated coordinates, we manually inspected the alignments to determine whether the SNP was located within a protein domain. We also identified the associated gene(s) and their Gene Ontology (GO) Annotations on FlyBase.

## 3. Results

### 3.1. Sequencing Results and Phylogenetic Analysis

All samples were successfully sequenced. All samples, regardless of pool size (e.g., 1, 5, or 20 mites), produced a relatively even number of reads both before (15,172,758 ± 515,109; average ± standard error (SE)) and after (9,915,847 ± 311,391) quality control measures were implemented (e.g., excluding reads with Phred quality <30 and duplicated reads). This read evenness was also prevalent within each infrapopulation both before and after quality control (Table S1). The best fit model of nucleotide substitution for the *cox1* ML phylogenetic analysis was TIM3+F+I+G4 according to AICc. As expected, all mites were identified as *Amerodectes protonotaria* (Figure S1).

### 3.2. Read Mapping and Variant Detection

**Figure 1:**
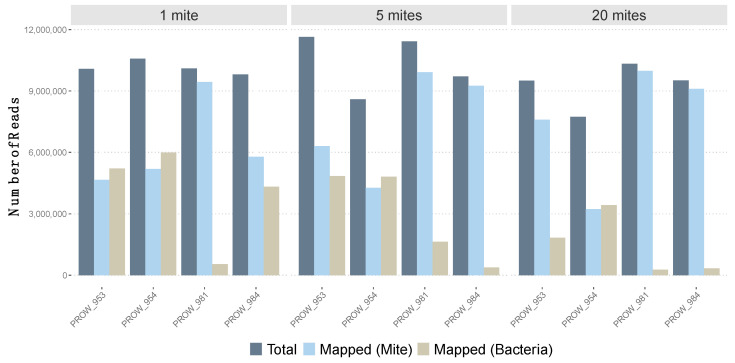
Number of total reads (after quality control, in dark blue) for each sample versus the number of mapped reads to both the feather mite genome reference (light blue) and three Burkholderiaceae bacterial genomes (tan).

Initial read mapping (i.e., before removing low-quality mapped reads) varied across samples using the feather mite reference genome and bacterial genomes (Figure 1). Using the feather mite reference genome, read mapping varied from 41.8% to 96.5% (average of 70.7% with SE of 6.5%). For three of the four one-mite (individually sequenced) samples, less than 60% of reads mapped the feather mite reference; the average across all four one-mite samples was 61.9% ± 10.9%. Average read mapping to the feather mite genome increased to 71.5 ± 11.5% with the five-mite pools and increased further to 78.5 ± 12.8% with the 20-mite pools. With an average of about 7 million reads mapped per sample, and given the genome size (59.6 Mb), this amounts to an average sequencing depth of 33X. Conversely, using the Burkholderiaceae genomes as a reference, read mapping varied from 2.6% to 56.6% across all samples (average: 28.6 ± 6.4%). Average read mapping to these bacteria generally decreased as pool sizes increased, from 39.4 ± 11.7% (1-mite samples) to 28.9 ± 12% (5-mite pools) to 17.4 ± 10% (20-mite pools). Only reads that passed the read mapping quality filter (i.e., mapping score > 20 to feather mite genome) were used for downstream SNP calling.

**Figure 2:**
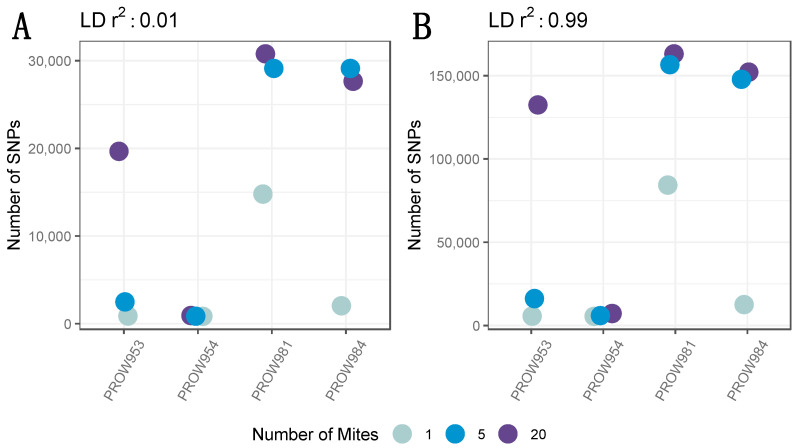
The number of SNPs across infrapopulations (labeled as the individual hosts) in which genotyping rate was not considered (i.e., all SNPs were included) with a linkage threshold of (**A**) 0.01 and (**B**) 0.99. Colors represent the number of mites in each pool and points are jittered around each individual host.

A total of 3,338,797 candidate genome-wide SNPs were initially identified prior to any filtering. After removing low-quality sites and sites different from the reference but identical to one another, a total of 2,735,601 candidate SNPs remained. A total of 2,706,989 SNPs remained after setting the MAF threshold to 0.05 and depth to 10X. Before removing missing genotype data, 41,108 SNPs remained with a linkage threshold of 0.01, whereas 250,142 SNPs remained with a linkage threshold of 0.99. The number of SNPs (and variation in the number of SNPs) differed both within and across infrapopulations prior to removing missing genotype data. Generally, the pattern followed expectations of the fewest SNPs in the one-mite samples, an intermediate number of SNPs in the five-mite samples, and the greatest number of SNPs in the 20-mite samples (Figure 2).

**Figure 3:**
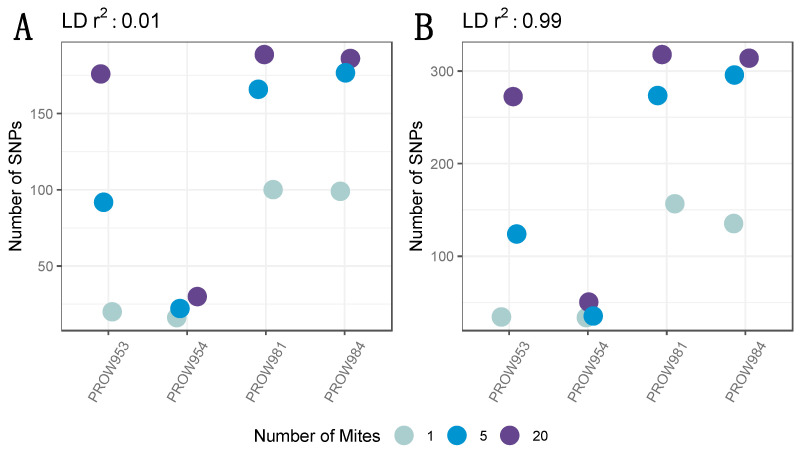
The number of SNPs across infrapopulations (labeled as the individual hosts) after filtering missing genotyping data with a linkage threshold of (**A**) 0.01 and (**B**) 0.99. Colors represent the number of mites in each pool and points are jittered around each individual host.

After filtering missing genotype data, only 737 SNPs remained. Implementing a conservative linkage threshold of 0.01 retained 235 SNPs. The variation in the number of SNPs per sample followed a similar pattern in the filtered dataset as the non-filtered dataset (Figure 3A). In other words, the number of SNPs increased with the number of individuals in the pool. While additional SNPs were retained using the more lenient linkage threshold of 0.99 (*n* = 439), the pattern of variation across samples was nearly identical to the dataset using a 0.01 linkage threshold (Figure 3B).

**Figure 4:**
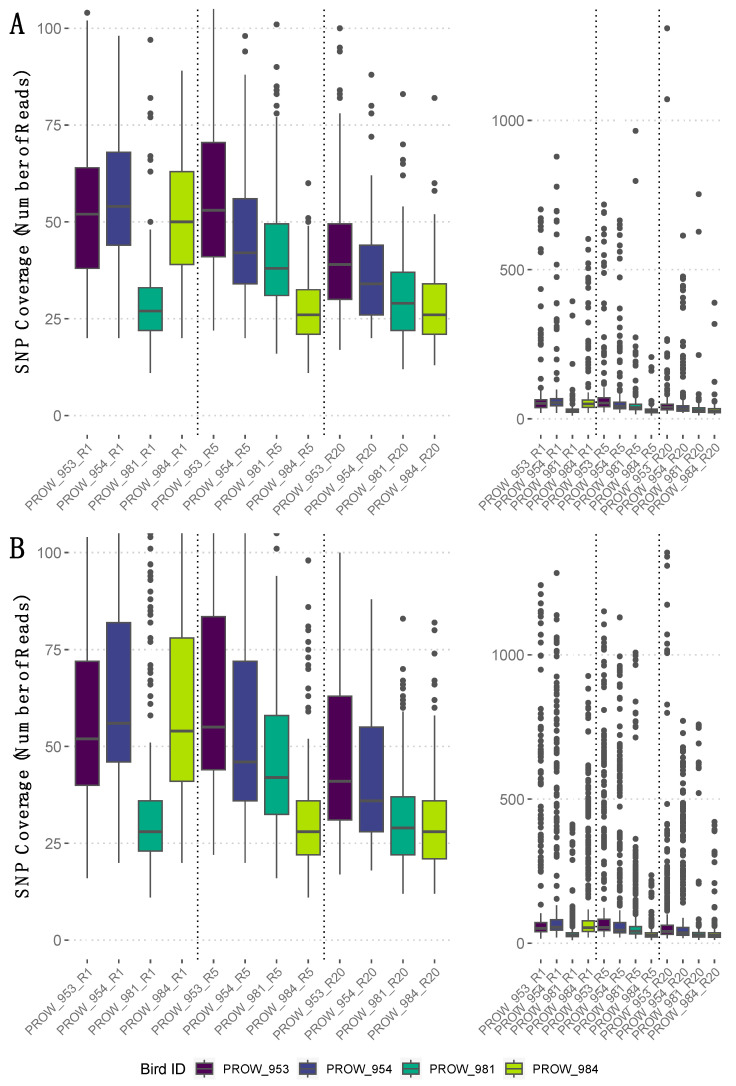
The number of reads that mapped to each SNP (i.e., SNP coverage) for the filtered dataset (i.e., no missing genotype data) per sample with a linkage threshold of (**A**) 0.01 and (**B**) 0.99. Rightmost panels show the full range of SNP coverage values per sample, whereas leftmost panels are enlarged and truncated versions of the rightmost panels to highlight the median and interquartile ranges of SNP coverage values per sample. Samples are ordered by the number of mites represented in each pool and divided by a dotted vertical line. Specifically, leftmost samples (i.e., those ending in “R1”) indicate one-mite samples, the middle four (“R5”) indicate five-mite samples, and the rightmost four (“R20”) indicate 20-mite samples. Darker colors represent hosts from Illinois and lighter colors represent hosts from Louisiana.

SNP coverage varied within and across samples (Figure 4, rightmost panels). Average SNP coverage for the filtered dataset (i.e., no missing genotype data) varied between linkage thresholds, but the median SNP coverage values were similar (Figure 4, leftmost panels). Specifically, the SNPs retained using a more conserved linkage threshold of 0.01 had an average coverage of 59X (median = 38X; Figure 4A), whereas the SNPs retained using a linkage threshold of 0.99 had an average coverage of 96X (median = 40X; Figure 4B).

### 3.3. Infrapopulation Genetic Structure and Diversity

**Figure 5:**
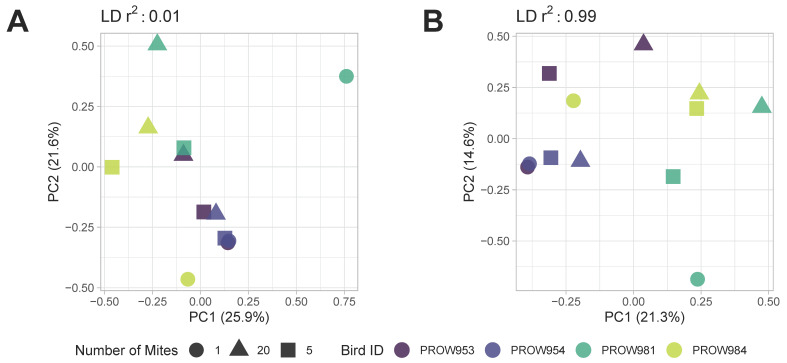
Principal components analysis (PCA) plots of *Amerodectes protonotaria* mites based on genome-wide SNPs prior to removing missing data with linkage thresholds of (**A**) 0.01 and (**B**) 0.99. Different pool sizes are represented by different shapes (circle = 1 mite; square = 5 mites; triangle = 20 mites) and colors represent individual hosts. Darker colors represent hosts from Illinois and lighter colors represent hosts from Louisiana.

**Figure 6:**
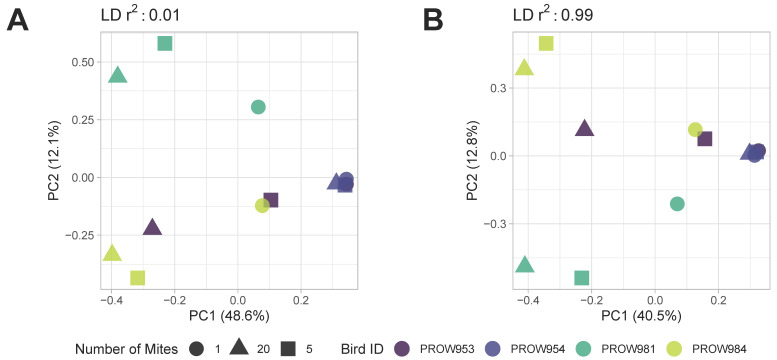
Principal components analysis (PCA) of *Amerodectes protonotaria* mites based on genome-wide SNPs after removing missing data with linkage thresholds of (**A**) 0.01 and (**B**) 0.99. Different pool sizes are represented by different shapes (circle = 1 mite; square = 5 mites; triangle = 20 mites) and different colors represent individual hosts. Darker colors represent hosts from Illinois and lighter colors represent hosts from Louisiana.

Both before (Figure 5) and after (Figure 6) removing missing genotype data, principal components analyses indicated that data were structured primarily by the individual bird from which the mites were collected (i.e., samples were primarily structured by infrapopulation). No obvious structure was seen by the number of mites in the pool or the location of collection. For the dataset in which missing data were retained, PC1 accounted for over 20% of the variation using both linkage thresholds, whereas PC2 accounted for 21.6% and 14.6% of the variation using linkage thresholds of 0.01 and 0.99, respectively (Figure 5). For the dataset in which missing genotype data were excluded, PC1 accounted for over 40% of the variation in the data and PC2 accounted for around 12% using both linkage thresholds (Figure 6).

**Figure 7:**
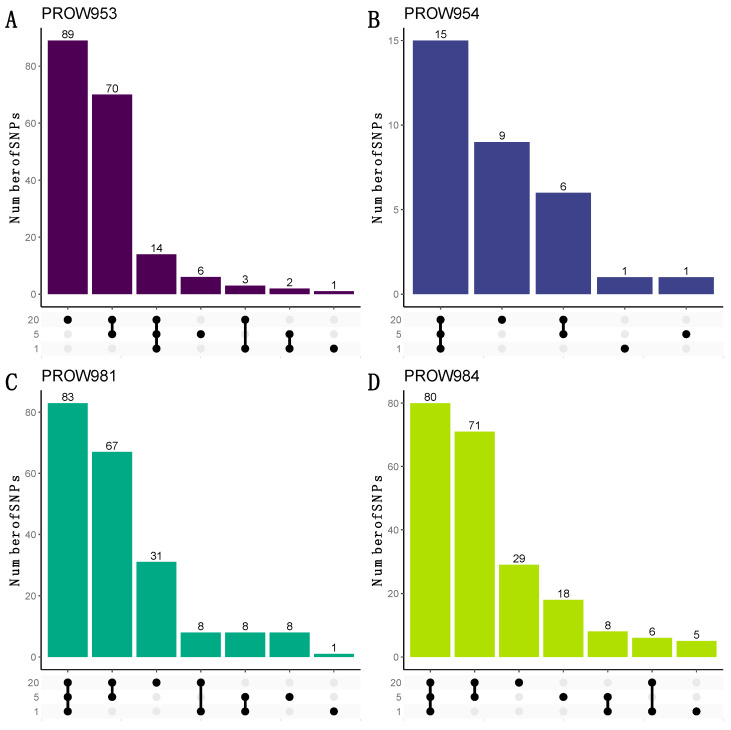
UpSet plots to visualize the number of shared and unique SNPs within and across sample scales from the dataset with a linkage threshold of 0.01. Rows represent different numbers of mites in each sample (20 mites, 5 mites, and 1 mite). Each column represents sets of SNPs that are either shared by samples (denoted by filled black dots connected with lines) or unique to a sample (denoted by black dots with no connections). Values on top of columns represent the size of the set. Each panel represents a different host individual, with the unique identifier as the panel title. Panels (**A**,**B**) were samples collected in Illinois and panels (**C**,**D**) were samples collected in Louisiana.

**Figure 8:**
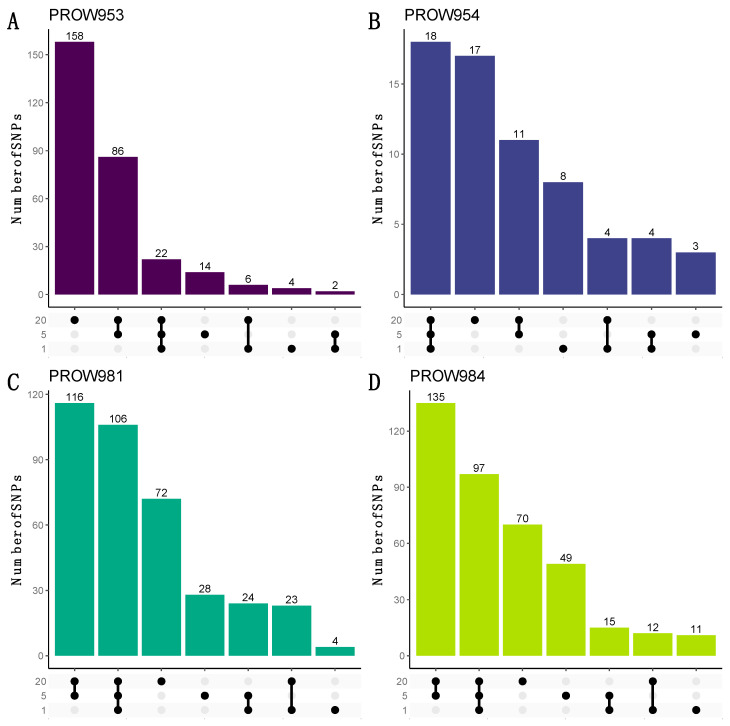
UpSet plots to visualize the number of shared and unique SNPs within and across sample scales from the dataset with a linkage threshold of 0.99. Rows represent different numbers of mites in each sample (20 mites, 5 mites, and 1 mite). Each column represents sets of SNPs that are either shared by samples (denoted by filled black dots connected with lines) or unique to a sample (denoted by black dots with no connections). Values on top of columns represent the size of the set. Each panel represents a different host individual, with the unique identifier as the panel title. Panels (**A**,**B**) were samples collected in Illinois and panels (**C**,**D**) were samples collected in Louisiana.

UpSet plots were used to illustrate how many and which SNPs were shared within and across infrapopulations. When using the more conservative linkage threshold of 0.01, the greatest number of the shared SNPs are shared at all scales for three out of the four hosts (one mite, five mites, and twenty mites; Figure 7B–D). For the remaining host (PROW953), most of the SNPs are unique to the 20-mite sample (Figure 7A). Results using a linkage threshold of 0.99 were slightly different; more SNPs were shared within only the five- and 20-mite samples as opposed to all three scales, except for host PROW954 (Figure 8). There were only six SNPs that were common to all four samples under both linkage thresholds.

We conducted BLASTx analyses of regions flanking the six SNPs that were common to all samples under both linkage thresholds to explore the potential function of the genes in which SNPs are located. We found that five of the six SNPs had BLASTx hits on the FlyBase database, and three of the six SNP sites had E-value scores less than 1 (Table 1). For those three SNP sites with E values less than 1, we identified the associated gene(s) and GO terms (Table 2). No SNPs were located within a protein domain.

## 4. Discussion

Microscopic symbionts present challenges in HTS studies. Nonetheless, we have demonstrated through pooled HTS that valuable information can be gleaned from sequencing individuals and pools of individuals from the same infrapopulation. Although both scenarios (individual sequencing and pooled sequencing) have advantages and disadvantages, our results demonstrate special technical and biological considerations to consider during the planning stages of HTS genomic studies of symbionts. Our data indicated that the downstream filtering and interpretation of data can also influence our understanding of coevolution at different evolutionary scales. Our results highlight both evolutionary and ecological insight into symbionts and their coevolutionary relationships with hosts that may have otherwise been overlooked by individual or pooled sequencing alone.

All samples, regardless of the number of individuals included in the pool, yielded a relatively even number of reads before and after implementing quality control measures (Table S1). This indicates that even an ultralow amount of tissue from an individual sample (e.g., a ~450 µm in length feather mite with ~0.5 ng of gDNA; A.E.M., personal observations) can be successfully sequenced in whole-genome shotgun sequencing projects using similar DNA extraction and library preparation protocols. This information can be important for future HTS studies on microscopic symbionts as well as the coevolutionary relationships they have with their hosts. However, the number of reads that mapped to the feather mite genome varied considerably within and across individual hosts (Figure 1). For three of the four single-mite samples, only half of the reads (51.4 ± 0.04% (SE)) mapped to the feather mite genome. This suggests that researchers who intend to conduct whole-genome HTS of individual microscopic symbionts, such as feather mites, may expect to discard approximately half of the raw reads at the mapping stage for most samples. However, in this study, we did not surface-sterilize mites prior to DNA extractions [66] to remove mite ectosymbionts (e.g., bacteria). Doing so may increase the number of feather mite reads while reducing the number of non-mite reads sequenced (see below). This knowledge may impact decisions on the type of sequencing technology to use or the number of samples that can be sequenced.

It may be expected that the relative abundance of bacteria, whether endosymbiotic (e.g., found in the gut), ectosymbiotic, or environmental, will scale proportionally with the number of mites in a pool. Contrary to this expectation, the number of target (i.e., feather mite) reads increased with pool size and off-target (i.e., discarded) reads generally decreased with pool size (Figure 1). The source of the discarded reads in this study primarily belonged to bacteria of the family Burkholderiaceae. A similar negative relationship between pool size and the relative abundance of bacteria has been observed in a multiplex PCR experiment of spider mites [67]. It is possible that the ultralow-input samples are at an inherently higher risk of contamination during wet lab procedures (e.g., DNA extraction, sonication, library preparation, sequencing; [68]). It is equally possible that bacterial symbionts vary in concentration among individual mites. In any case, for microscopic organisms, it is important to account for reads that do not map to the target genome to distinguish biological (e.g., horizontal gene transfer [69]) from non-biological (e.g., true contaminants [70]) signals. There are several well-established strategies and tools to determine target from off-target DNA for HTS data [71,72,73,74].

The SNP filtering criteria can be a critical component to identify genomic variants and interpret results [75]. Therefore, we explored datasets generated using different filters. Our data indicated that the number of SNPs differed between datasets (e.g., including or excluding missing data, contrasting linkage thresholds). Despite these differences, the overall patterns between and within samples remained nearly identical (Figure 2 and Figure 3). This result demonstrates that the overall patterns are robust to different filtering criteria. Namely, the number of SNPs generally increases with the number of individuals in the pool, regardless of how the data are filtered. One infrapopulation, PROW954, did not deviate from this pattern, but we found much fewer SNPs within this infrapopulation (across all pool sizes) than the others. It is unclear why the genetic diversity of the mites from this infrapopulation is so uniquely low but highlights the variation of evolutionary processes occurring at the infrapopulation-level in host–symbiont systems. We also found that enough SNPs remained at a high enough coverage in the 5- and 20-mite samples after filtering (Figure 4) to conduct downstream population genetic analyses, suggesting that genetic diversity is proportional to the pool size and possibly infrapopulation size, although this was not directly tested here.

These results are consistent with previous feather mite studies, which demonstrated that mitochondrial DNA genetic diversity within infrapopulations can be significant [35] and that genetic diversity increases with infrapopulation sizes [27]. Whether or not there is an asymptote of diversity within an infrapopulation (i.e., how much genetic diversity can one more individual contribute to the infrapopulation?) requires more replication and potentially larger pool sizes. This insight can help us fill basic biological knowledge gaps of mite sexual reproduction behaviors (e.g., inbreeding [76]) and transmission (e.g., immigration [77]). Sequencing male and female mites separately (individually or pooled) may also help to answer sex-specific genomic questions of many types of avian mites, such as sex-based variation in genome size and ploidy [78,79,80]. These results may also have important implications for coevolutionary studies of hosts and symbionts. For example, sequencing only one individual symbiont from an infrapopulation may underestimate the genetic diversity of the symbionts on an individual host (depending on the life history of the symbiont [81]). This underestimation may then lead to a misinterpreted coevolutionary signal. However, with carefully designed sampling and a large enough sample size, these risks can be minimized [82].

Although the number of SNPs varied with pool size within infrapopulations, we found that infrapopulations were primarily structured by the individual host more so than pool size (Figure 6). This pattern, like the patterns seen with the number of SNPs, was also consistent when missing genotype data were included (Figure 5). These results indicate that the mites collected from an infrapopulation (i.e., a single feather from a single bird) are more closely related to each other than to another infrapopulation, similar to what was observed in Virrueta Herrera et al.’s work [82], in which two lice per freshwater seal host were individually sequenced. These results indicate that infrapopulation-level evolutionary processes are driving these general patterns of structure rather than technical choices like pool size. UpSet plots also suggest a similar pattern such that most SNPs identified within an infrapopulation are shared by all three pool sizes (Figure 7B–D). In other words, most SNPs are not unique to specific pool sizes, which further supports a biological signal of infrapopulation relatedness. This also indicates that it is possible to detect SNPs of high confidence (i.e., SNPs that were identified from one-mite samples) in pools of 5 or 20 individuals. Thus, we can be highly confident in the SNPs identified from pooled samples, especially those shared with the one-mite samples. With a more relaxed linkage threshold (Figure 8), we observed the pattern shift for samples collected in Louisiana. Most SNPs were shared only between the five- and 20-mite samples (i.e., not all three pool sizes). This is not largely unexpected because pooled samples potentially contribute redundant information in clustered genomic regions compared to individual samples [83]; thus, they are more likely to have linked SNPs. Despite these differences, these patterns still suggest that most SNPs are not unique to specific pool sizes. Ideally, each sample will contain an equal number of individuals so that each pool contains an equal amount of DNA and an equal representation of individuals [18]. However, our results suggest that enough similarity exists within infrapopulations (Figure 7) and enough diversity exists between infrapopulations (Figure 6) that if an equal number of individuals cannot be obtained for every sample (for example, due to difficulty of collection, rarity of the species, or variation in infrapopulation sizes), signals of (infra)population differentiation can still be detected.

It is possible that some SNPs are located on genes that are important for mite fitness (e.g., survival and reproduction), which enables mites to quickly adapt to, persist on, and maintain a symbiotic relationship with avian hosts. We preliminarily tested if our pooled data could be used for such analyses. Two of the three FlyBase BLASTx hits with E-values less than 1 (Table 1) were associated with specific genes annotated for *Drosophila melanogaster* (Table 2). First, according to FlyBase, metabotropic GABA-B-R2 enables G protein-coupled GABA-B receptor activity and has several biological functions, such as a response to mechanical stimuli and olfactory behaviors. One mechanical pressure that feather mites experience is related to their need for permanent attachment to flight feathers; they must remain attached without being dislodged, even while the bird is in flight. Adaptive behavioral responses to changing turbulence and other dynamic conditions would certainly increase their chances of survival on hosts. Feather mites also encounter a large number of food resources on feathers [84] and may eat a diverse array of those available resources [85]; the ability to sense and adapt to novel food resources may also increase survival. Second, according to FlyBase, the male fertility factor kl5 is involved in sperm motility, which could significantly impact mite reproduction or sexual selection. Future investigations on these genomic regions could help clarify the important role these SNPs may play in mite fitness. Alternatively, exploring variants in genomic regions that are known to be involved in coevolutionary processes (e.g., [86,87,88]) could be valuable to our understanding of how host–symbiont interactions are maintained or change over time. However, these specific regions are not yet known for this system.

## 5. Conclusions

Our feather mite population genomics study highlights the advantages and disadvantages of applying HTS for individual-level sequencing versus infrapopulation-level pooled sequencing of microscopic symbionts in coevolutionary studies. Trade-offs exist with respect to individual haplotype information capture (individual sequencing) or loss (pooled sequencing) as well as an increase (pooled sequencing) or decrease (individual sequencing) in read mapping. Pooled sequencing is more likely to capture “hidden” genetic variants that exist in an infrapopulation compared with individual sequencing, but because infrapopulation diversity seems to be structured by the individual host regardless of pool size in this system, a loss of this information at the individual level may not impact biological inferences. Based on our results, a balanced strategy of sequencing multiple individuals per infrapopulation may be ideal. This approach would confidently capture haplotype information while not overlooking potentially informative infrapopulation genetic diversity and would ultimately improve our understanding of host–symbiont coevolution at multiple evolutionary scales.

## Figures and Tables

**Table 1:** FlyBase BLASTx results of 100 bp regions flanking the six SNPs (listed as contig:position) common to all samples under both linkage thresholds.

SNP	Polypeptide	Score	E Value	FlyBase ID (Polypeptide)	Length (aa)
866:1415	GABA-B-R2-PC	31.5722	0.173236	FBpp0308298	1224
	GABA-B-R2-PB	31.5722	0.173236	FBpp0083566	1221
	GABA-B-R2-PA	31.5722	0.173236	FBpp0083565	1220
1716:115	mamo-PC	25.7942	9.50577	FBpp0311886	799
1885:6843	CG32102-PA	30.4166	0.385929	FBpp0075710	88
2045:5306	Mp-PS	26.5646	5.57281	FBpp0308935	804
	Mp-PR	26.5646	5.57281	FBpp0301686	1039
2173:2309	NA	NA	NA	NA	NA
2301:4976	kl-5-PC	33.8834	0.0349058	FBpp0312367	4559

**Table 2:** Gene Ontology (GO) annotations for the three SNP (listed as contig:position) regions with E values less than 1 from the FlyBase BLASTx results.

SNP	Associated Gene	Annotation Symbol	FlyBase ID (Gene)	Molecular			Biological							Cellular					
866:1415	metabotropic GABA-B receptor subtype 2	CG6706	FBgn0027575	X		X	X	X		X	X	X	X		X	X		X	
1885:6843	CG32102	CG32102	FBgn0052102										X						
2301:4976	male fertility factor kl5	CG45786	FBgn0267433		X	X			X				X	X			X	X	X
				receptor	small molecule binding	other molecular function	cell organization/biogenesis	transport/localization	reproduction	behavior	response to stimulus	signaling	other biological process	cytoskeleton	membrane	cell periphery	cell projection	macromolecular complex	other cellular component

## Data Availability

The data presented in this study are available in Table S1 or are available upon request from the corresponding author. All bioinformatic pipelines and analytical scripts are available on GitHub (https://github.com/alixmatthews/protonotaria_pool_experiment) (accessed on 6 October 2023).

## Supplementary Materials

The following supporting information can be downloaded at: https://www.mdpi.com/article/10.3390/life13102054/s1, Table S1: Sample information.; Figure S1: Maximum likelihood (ML) phylogeny estimated from the cytochrome c oxidase subunit 1 (cox1) barcoding gene using a curated dataset of parulid *Amerodectes*, *Tyrannidectes*, *Proctophylllodes*, and *Trouessartia* feather mite lineages. *Amerodectes protonotaria* samples are highlighted in yellow with the 12 samples from the present work ending in “pw” at the branch tips. Values at nodes indicate ML ultrafast bootstrap (BS) support values for each mite lineage; nodes with >99 BS values are indicated by asterisks (*). The scale bar represents nucleotide substitutions per site.
