# Supplementary material for: Population Genomics of Pooled Samples: Unveiling Symbiont Infrapopulation Diversity and Host–Symbiont Coevolution
Source: Life (Basel). 2023 Oct 14;13(10):2054. doi: 10.3390/life13102054 (PMC10608719; doi:10.3390/life13102054)
Supplement: Supplementary file 1 [file life-13-02054-s001.zip › Figure S1.pdf]

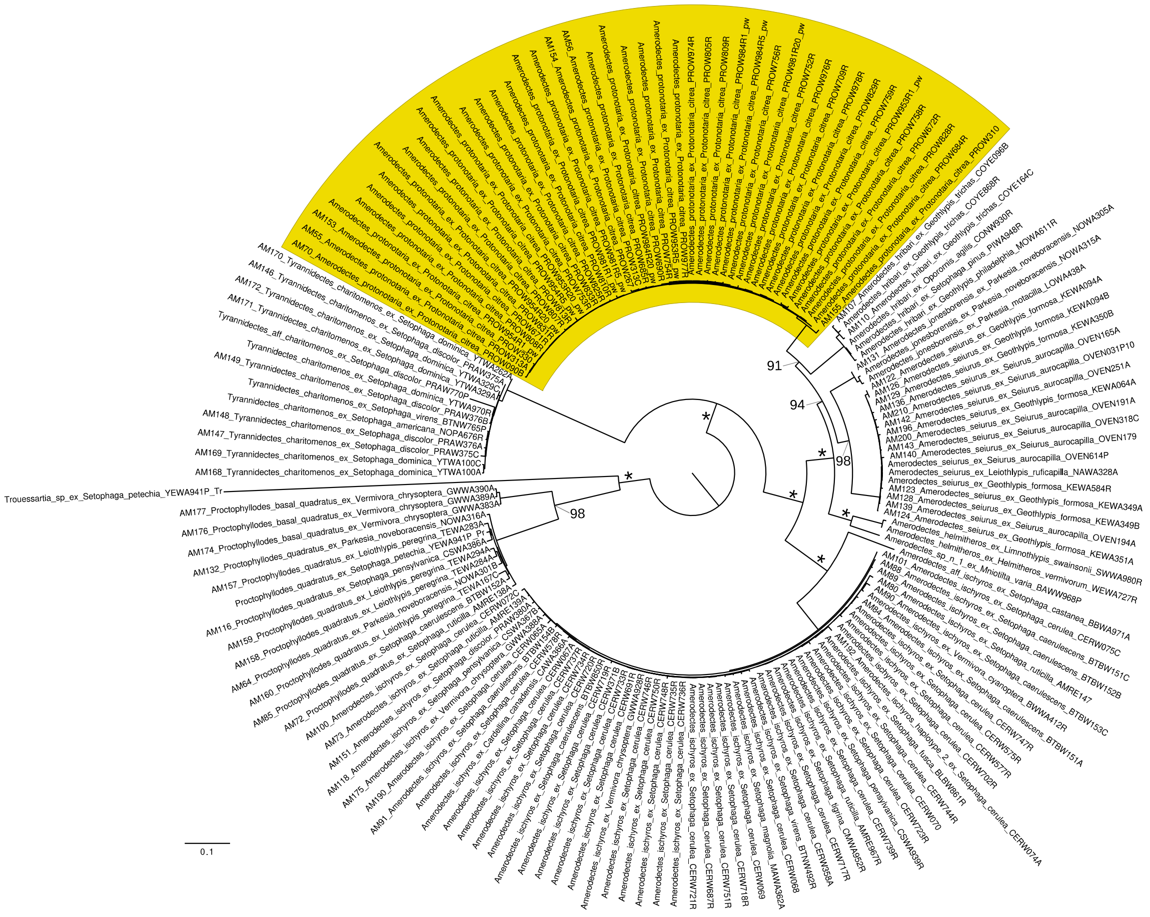

Figure S1. Maximum likelihood (ML) phylogeny estimated from the cytochrome c oxidase subunit 1 (*cox1*) barcoding gene using a curated dataset of parulid *Amerodectes*, *Tyrannidectes*, *Proctophylllodes*, and *Trouessartia* feather mite lineages. *Amerodectes protonotaria* samples are highlighted in yellow with the 12 samples from the present work ending in “pw” at the branch tips. Values at nodes indicate ML ultrafast bootstrap (BS) support values for each mite lineage; nodes with >99 BS values are indicated by asterisks (*). The scale bar represents nucleotide substitutions per site.
